# Developmental analyses of divarications in leaves of an aquatic fern *Microsorum pteropus* and its varieties

**DOI:** 10.1371/journal.pone.0210141

**Published:** 2019-01-25

**Authors:** Saori Miyoshi, Seisuke Kimura, Ryo Ootsuki, Takumi Higaki, Akiko Nakamasu

**Affiliations:** 1 Department of Bioresource and Environmental Sciences, Faculty of Life Sciences, Kyoto Sangyo University, Kyoto, Japan; 2 Center for Ecological Evolutionary Developmental Biology, Kyoto Sangyo University, Kyoto, Japan; 3 Department of Natural Sciences, Faculty of Arts and Sciences, Komazawa University, Tokyo, Japan; 4 Faculty of Chemical and Biological Sciences, Japan Women's University, Tokyo, Japan; 5 International Research Organization for Advanced Science and Technology, Kumamoto University, Kumamoto, Japan; 6 Meiji Institute for Advanced Study of Mathematical Sciences, Meiji University, Tokyo, Japan; University of Western Sydney, AUSTRALIA

## Abstract

Plant leaves occur in diverse shapes. Divarication patterns that develop during early growths are one of key factors that determine leaf shapes. We utilized leaves of *Microsorum pteropus*, a semi-aquatic fern, and closely related varieties to analyze a variation in the divarication patterns. The leaves exhibited three major types of divarication: no lobes, bifurcation, and trifurcation (i.e., monopodial branching). Our investigation of their developmental processes, using time-lapse imaging, revealed localized growths and dissections of blades near each leaf apex. Restricted cell divisions responsible for the apical growths were confirmed using a pulse-chase strategy for EdU labeling assays.

## Introduction

Plants are mainly consisted of stems, roots, and leaves. The leaves are critical for photosynthesis and vary widely in size and shape, although they all develop similarly from a small group of cells, called leaf primordia, which locate on shoot apical meristems. Mathematical models have been used to understand complex natures of leaf-shape formation [1], [2], [3], [4]. Developmental patterns in leaf primordia that determine leaf shapes are highly diverse among species [5]. Particularly, a diversity of cell-division sites in leaf primordia can be observed in developmental stages of different plant species with simple or compound leaves [6], [7], [8]. There are four major types of polarity in the growth patterns along longitudinal axes in simple leaves [9]: acropetal, basipetal, bidirectional (divergent), and diffuse growths (with no apparent allometry). The difference may determine initiation positions of leaf appendage in more complex leaves as mentioned in [10]. Then it could be result in formations of characteristic leaf shape.

Divarication pattern (two-dimensional branching) is one of key factors that determine leaf shapes. A variation in leaf divarications can be classified into three major types: no lobes, bifurcation (or fused leaf) and monopodial trifurcation, the last is common among plants with compound or dissected leaves. Leaf bifurcations are rarely observed among Tracheophyta and they are limited to some plants, such as ferns, and lamina of some seaweeds [11]. Mechanisms that cause the leaf-blade bifurcations may be common among such plants. Almost all fern leaves have coiled axes (crosiers) in their early developmental stages, as a consequence of abaxial–adaxial disparities in their growth patterns [12], which make it difficult to study the developmental processes of fern leaves.

*Microsorum pteropus* [13], [14], a semi-aquatic, epiphytic fern, has leaves that do not tightly coil in any stages of their developments. In addition, the fern has many varieties, which exhibit the different types of leaf divarication. To examine a variation in their distal growth patterns, we used time-lapse images to analyze the growth processes in the leaves. We used a replica method to observe small-and-simple shapes of epidermal cell at each distal end of the glowing leaves in the *Microsorum* cultivars. We subsequently confirmed cell divisions only at the distal part(s) of the leaves, using pulse-chase experiments for assays using EdU of thymidine analog assays.

## Materials and methods

### Plant cultivation

*M*. *pteropus* wild type and its six varieties were used in the present study. The plants were grown in a room with a continuous light condition at 22°C. The plants were grown on wet soil in a plastic dish from times of differentiation of adventitious bud to nurse plant stages. Subsequently the ferns were replanted in soil in Magenta box culture boxes.

### Molecular phylogenetic analysis

Genomic DNAs were extracted from leaves of the *M*. *pteropus* cultivars using a DNeazy plant mini kit (Quiagen, Dutch). The diluted DNAs (20 μL) were amplified with 30 to 40 PCR cycles (94°C for 1.5 min, 55°C for 30 s, and 7°C for 2 min) using a homemade Taq polymerase. We used three chloroplast genome regions (except *rps4-trnS* IGS), as described in a study by Kreier *et al*. (2008) [15]. The regions include a non-coding region (*TrnL-F*) and the following two coding regions: (1) a large subunit of a ribulose-bisphosphate carboxylase (*rbcL*) and (2) a region similar to a ribosomal protein small subunit 4 (*rps4*). The primer arrays are presented in Table 1. Obtained PCR products were purified using a Gel Extraction Kit (Quiagen, Dutch) or an Illustra ExoProStar (GE Healthcare UK Ltd.). The constructs were subsequently sequenced by FASMAC Co. Ltd. (Kanagawa).

**Table 1:** Sequences of primer utilized in a phylogenetic analysis.

**rbcL univ aF**	ATGTCACCACAAACAGAGACTAAAGC
**rbcL univ cR**	GCAGCAGCTAGTTCCGGGCTCCA
**trnL-F B49873**	GGTTCAAGTCCCTCTATCCC
**trnL-F B49873**	ATTTGAACTGGTGACACGAG
**rps4 fpr micF**	AAAATACCCAATTTGGGAGAA
**rps4 fpr micR**	TGATTTAGATTCTGTTCCAAAC

Three arrays were used for a molecular phylogeny analysis: *rbcL*, *trnL−F*, and *rps4*.

The obtained sequences were analyzed using GENETYX-MAC version 18 (GENETYX, Tokyo). The DNA sequences of each plant were combined in the following order: *trnL-F*, *rbcL*, and *rps4* [15], [16], and then aligned. Non-identical regions within each population were removed. Phylogenetic trees were constructed using the neighbor-joining method with a bootstrap test of 5000 replicates using MEGA software version 4 (www.megasoftware.net). Sequences of reference for *M*. *pteropus* and other ferns were obtained from GenBank (Table 2). The sequences obtained from our analysis were deposited in GenBank (Table 3).

**Table 2:** Accession numbers of plant species utilized in a phylogenetic analysis.

Species	*rbcL*	*rps4*	*trnL-F*
*Belvisia annamensis* (C. Chr.) S.H. Fu	EU482931	EU482976	EU483025
*Belvisia mucronata* (Fée) Copel.	AY362562	AY362629	DQ642232
*Belvisia platyrhynchos* (Kunze) Copel.	DQ642152	DQ642190	DQ642233
*Drymotaenium miyoshianum* (Makino) Makino	AY362563	AY362630	DQ179640
*Goniophlebium argutum* (Wall. ex Hook.) J. Sm. ex Hook.	DQ164442	DQ164473	DQ164505
*Goniophlebium formosanum* (Baker) Rödl-Linder	AB043100	AY096224	DQ642235
*Goniophlebium mehibitense* (C. Chr.) Parris	EU482932	EU482977	EU483026
*Goniophlebium niponicum* (Mett.) Bedd.	ABO43098	AY362626	EU483027
*Goniophlebium persicifolium* (Desv.) Bedd.	EU482933	AY096225	EU483028
*Goniophlebium pseudocommutatum* (Copel.) Copel.	EU482934	Eu482978	EU483029
*Goniophlebium subauriculatum* (Blume) C.Presl	AF470342	DQ168812	AY083645
*Lecanopteris balgoyii* Hennipman	AF470328	EU482980	AY083631
*Lecanopteris carnosa* Blume	AF470322	AY096227	AY083625
*Lecanopteris celebica* Hennipman	AF470323	EU482981	AY083626
*Lecanopteris crustcea* Copel.	AF470329	EU482982	AY083632
*Lecanopteris luzonensis* Hennipman	AF470325	EU482983	AY083628
Species	*rbcL*	*rps4*	*trnL-F*
*Lecanopteris mirabilis* (C. Chr.) Copel.	AF470330	EU482984	AY083633
*Lecanopteris sarcopus* (Teijsm. & Binn.) Copel.	EU482935	EU482985	EU483030
*Lecanopteris sinuosa* (Hook.) Copel.	AF470321	AY362634	AY083624
*Lemmaphyllum accedens* (Blume) Donk ex. Holttum	EU482936	EU482986	EU483031
*Lemmaphyllum carnosum* (J. Sm. ex Hook.) C. Presl	AF470332	AY362631	AY083635
*Lemmaphyllum diversum* (Rosenst.) Tagawa	EU482937	EU482987	EU483032
*Lemmaphyllum microphyllum* C. Presl ▲	EU482938	EU482988	EU483033
*Lepidogrammitis diversa* (Rosenst.) Ching	EU482939	EU482989	EU483034
*Lepisorus clathratus* (C.B. Clarke) Ching	DQ642154	DQ642192	DQ642236
*Lepisorus excavatus* (Willd.) Ching	DQ642155	DQ642193	DQ642237
*Lepisorus kawakamii* (Hayata) Tagawa	EU482940	EU482990	EU483035
*Lepisorus longifolius* (Bl.) Holtt.	DQ642157	DQ642195	DQ642239
*Lepisorus macrosphaerus* (Baker) Ching	EU482941	EU482991	EU483036
*Lepisorus megasorus* (C.Chr.) Ching	DQ642158	DQ642196	DQ642240
*Lepisorus monilisorus* (Hayata) Tagawa	EU482942	EU482992	EU483037
*Lepisorus pseudo-ussuriensis* Tagawa	EU482943	EU482993	EU483038
*Lepisorus thunbergianus* (Kaulf.) Ching	U05629	AY096226	DQ642241
*Lepisorus waltonii* (Ching) S.L. Yu	EU482944	EU482994	EU483039
*Leptochilus cantoniensis* (Baker) Ching	EU482945	EU482995	EU483041
*Leptochilus decurens* Blume	AY096203	AY096228	DQ179640
*Leptochilus cantoniensis* (Baker) Ching	EU482945	EU482995	EU483041
*Leptochilus decurrens* Blume ▲	AY096203	AY096228	DQ179640
*Leptochilus digitatus* (Baker) Noot.	EU482948	EU482998	EU483044
*Leptochilus elliptica* (Thunb.) Ching	EU482949	EU482999	EU483045
*Leptochilus hemionitideus* (Wall. ex C. Presl) Noot.	U05612	EU503044	EU503045
*Leptochilus hemitoma* (Hance) Ching	EU482951	EU483001	EU483047
*Leptochilus henryi* (Baker) Ching	EU482952	EU483002	EU483048
*Leptochilus simplifrons* (H. Christ) Tagawa	EU482953	EU483003	EU483049
*Leptochilus macrophyllus* (Blume) Noot. var. wrightii (Hook. & Baker) Noot.	EU482954	EU483004	EU483050
*Microsorum commutatum* (Bl.) Copel. ▲	AY362571	EU483005	EU483051
*Microsorum cuspidatum* (D. Don) Tagawa	AF470335	AY096230	AY983638
*Microsorum grossum* (Langsd. & Fisch.) S.B. Andrews ▲	EU482956	EU483007	EU483053
*Phymatosorus hainanensis* (Noot.) S.G.Lu	EU482960	EU483011	EU483059
*Microsorum insigne* (Blume) Copel.	EU482957	EU483008	EU483054
*Microsorum lastii* (Baker) Tardieu	EU482961	EU483012	EU483058
*Microsorum linguiforme* (Mett.) Copel.	AF470334	AY362635	AY083637
*Microsorum membranaceum* (D.Don) Ching	EU482962	EU483013	EU483059
*Microsorum membranifolium* (R.Br.) Ching	DQ642161	DQ642200	DQ642245
*Microsorum musifolium* (Blume) Copel.	AF470335	AY362636	AY083636
*Microsorum novo-zealandiae* (Baker) Copel.	DQ401116	DQ401126	DQ401121
*Microsorum papuanum* (Baker) Parris	DQ642162	EU483015	DQ642246
*Microsorum pteropus* (Blume) Copel.	EU482965	EU483016	EU483061
*Microsorum punctuatum* (L.) Copel.	DQ164444	DQ164475	DQ164508
Species	*rbcL*	*rps4*	*trnL-F*
*Microsorum pustulatum* (G. Forst.) Copel. ▲	DQ401117	DQ401127	DQ401122
*Microsorum scandens* (G. Forst.) Tindale	DQ401118	DQ401128	DQ401123
*Microsorum scolopendrium* (Burm.f.) Copel. ▲	DQ642163	DQ642201	DQ642247
*Microsorum spectrum* (Kaulf.) Copel. ▲	EU482967	EU483018	EU483064
*Microsorum thailandicum* T. Booknerd & Noot.	EU482969	EU483020	EU483066
*Microsorum varians* (Mett.) Hennipman & Hett. ▲	AY362566	AY362638	DQ179643
*Microsorum viellardii* (Mett.) Copel.	DQ179635	DQ179638	DQ179645
*Microsorum whiteheadii* A.R. Sm. & Hoshiz.	EU482970	EU483021	EU483067
*Microsorum zippelii* (Blume) Ching	AB23241	DQ642203	DQ642249
*Microsorum superficiale* (Blume) Bosman	EU482971	EU483022	EU483062
*Neocheiropteris palmatopedata* (Baker) H.Christ	AY362567	AY362640	DQ212059
*Neolepisorus phyllomanes* (H. Christ) Ching	EU482973	EU483024	EU483069
*Thylacopteris papillosa* (Blume) Krause ex J.Sm.	AY459174	AY459188	AY459183
*Pyrrosia polydactyla*	KY064512	DQ164502	DQ164530
*Platycerium stemaria*	EF463257	DQ164489	DQ164522

▲ symbols indicate representative fern species selected to generate second phylogenetic tree.

**Table 3:** Accession numbers of plant species obtained in this paper.

Species	*rbcL*	*rps4*	*trnL-F*
*Microsorum pteropus var*. *windelov*	LC322102	LC325240	LC325246
*Microsorum pteropus ‘Giagantia’*	LC322103	LC325241	LC325247
*Microsorum pteropus ‘Tropica’*	LC322104	LC325242	LC325248
*Microsorum sp*. *‘Thunder leaf’*	LC322105	LC325243	LC325249
*Microsorum sp*. *‘Fork leaf’*	LC322106	LC325244	LC325250
*Microsorum sp*. *‘tridentleaf’*,	LC322107	LC325245	LC325251

### Time-lapse imaging

Nurse plants were placed on wet soil, pushed against the wall of a Magenta box, and they were covered with a piece of wet paper (without trapping air bubbles) and maintained at 22°C within a bio-multi incubator (LH-80WLED-6CT, Nippon Medical & Chemical Instruments Co., LTD, Osaka). Images (x20 magnification) were taken every six hours for two months using a USB digital microscope (Dino Lite Pro LWD, AnMo Electronics Corporation, Taiwan). This equipment was placed on a silicon sheet to eliminate vibrations.

### Observation of epidermal cells

Epidermal cells were observed using a replica method, as follows. Each cut leaf was wiped with a paper towel and mixed dental paste was applied to both sides of the leaf. After solidifying, the pastes were removed from the leaf, creating negative molds. After clear nail polish that was put on the mold form or directly applied to the leaf was dried, it was taken off, put on a glass slide, and then flattened with a glass slide cover. Pictures of the positive (or negative) molds were taken using an upright microscope. From the pictures, sizes of epidermal cell were measured using ImageJ software (https://imagej.nih.gov/ij/).

### Detection of cell divisions

EdU-labeling assays were performed using a pulse-chase methods [9], [17], [18] to avoid signals obtained by endoreduplications, wherein cell cycles skip the mitotic phases [19]. We removed trichomes to enhance visualization of cell proliferation. Numerous trichomes (on both sides of leaf surface) were rubbed off with a glass capillary chilled in liquid nitrogen. the leaves (with trichomes removed) were immersed in a 10 μM EdU solution (Click-iT EdU Microplate Assay kit, Invitrogen, Japan) and allowed to grow for 1–2 days. The leaves were subsequently transferred to a normal water and allowed to grow for 8–16 hours until cell divisions became evident. Trichomes were removed again, and the leaves were immersed in a 90% ice-cold acetone for 10 minutes. They were washed with a phosphate buffered salts (PBS) and subsequently fixed with a formalin-acetic acid-alcohol (FAA), as outlined by Nakayama *et al*. 2015 [20]. The samples were washed two times for 5 min with 0.5% TritonX in PBSs, washed twice again with PBSs, and then, immersed for 1 hour (or 1.5 hours) under a dark condition in a reaction cocktail (Click-iT EdU Microplate Assay kit) prepared at the time of use. Subsequently, the leaves were rinsed two times for 20 minutes with PBSs. The samples were mounted on a glass slide, the abaxial side up, and observed under a fluorescent microscope (Nikon ECLIPSE 80i or OLYMPUS BX53F). Pictures were taken through the microscope’s lens.

## Results

### Leaf morphology and a molecular phylogenetic analysis of *Microsorum pteropus* and its varieties

**Fig 1:**
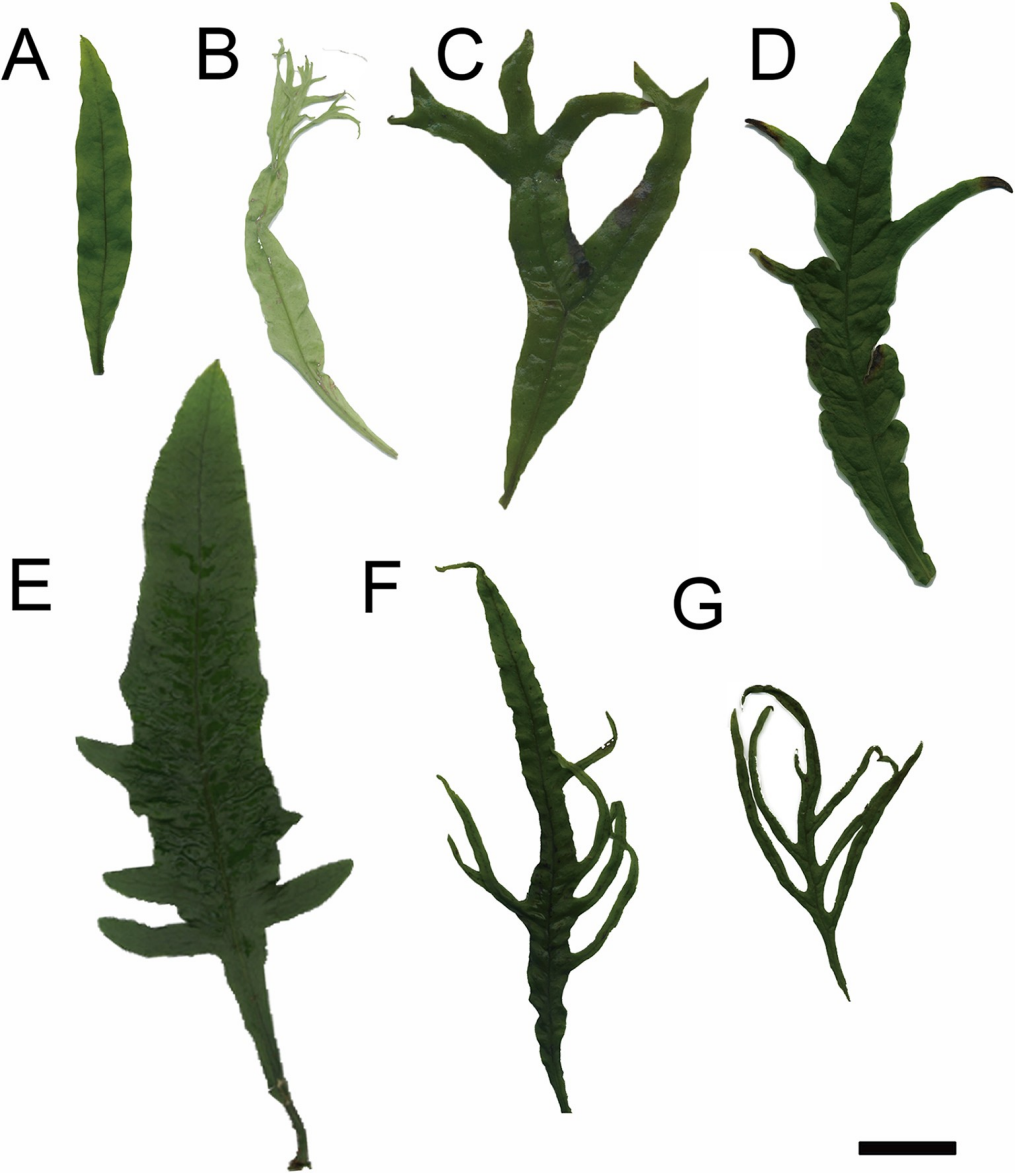
Leaf divarications observed in *M*. *pteropus* and its varieties. (A) Wild type (wt) of *M*. *pteropus*, varieties with bifurcated leaves; (B) *M*. *pteropus* var. *windelov* and (C) *M*. *pteropus ‘Gigantea’*, and varieties with monopodial leaves; (D) *M*. *pteropus ‘Tropica’*, (E) *Microsorum sp*. *‘Thunder leaf’*, (F) *Microsorum sp*. *‘Fork leaf’*, and (G) *Microsorum sp*. *‘tridentleaf’*. A scale bar represents 2 cm.

**Fig 2:**
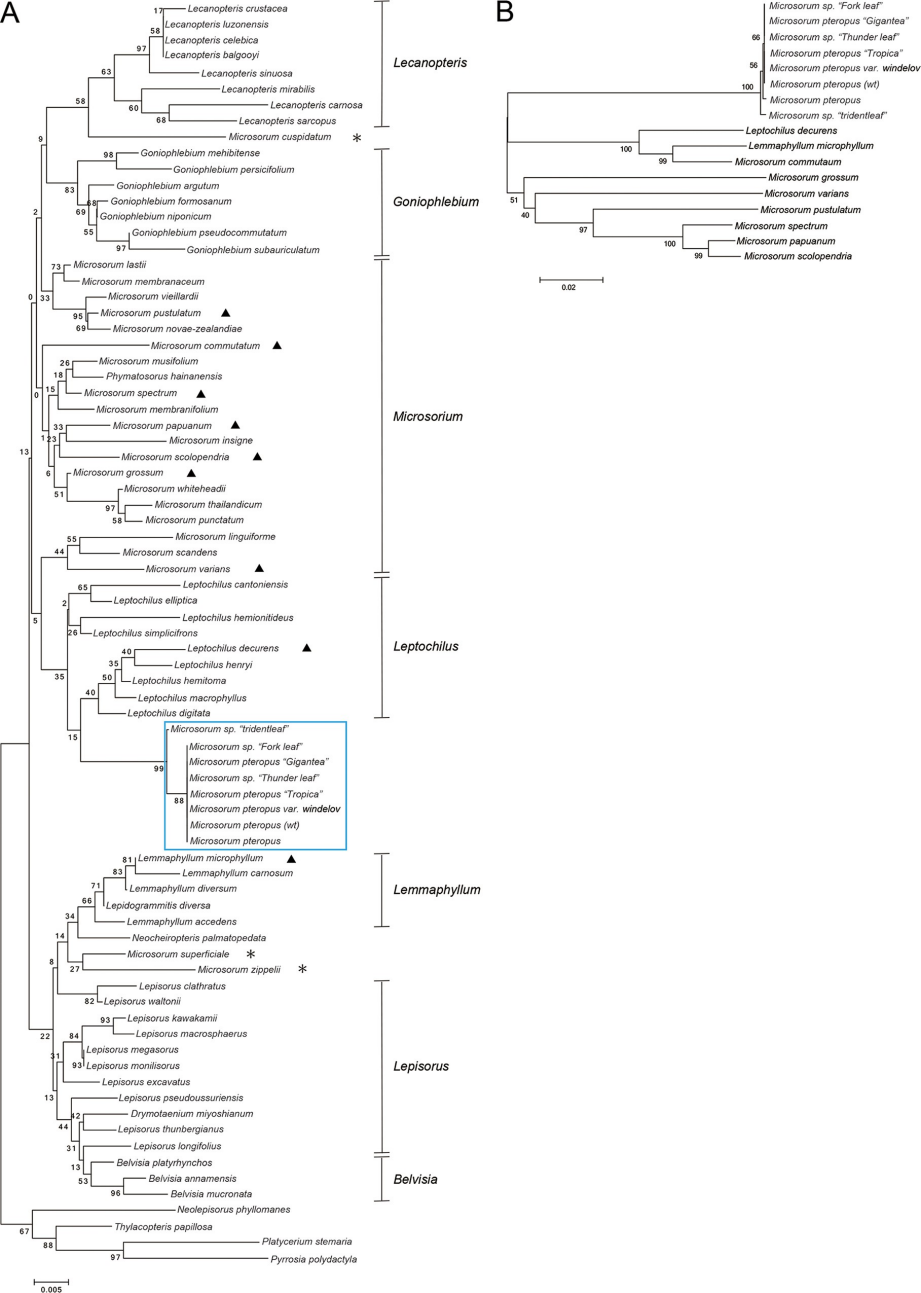
Phylogenetic relationships among the *M*. *pteropus* cultivars and other fern species. Phylogenetic tree constructions of (A) *M*. *pteropus*, its varieties and other fern species, and (B) the *M*. *pteropus* cultivars and representative fern species selected from (A) or Table 2 (denoted with triangles). The examined varieties classified into one group, are framed in a rectangle, then *Microsorum* radicals are indicated by asterisks in (A).

*Microsorum pteropus* possesses many varieties, which exhibit a variety of leaf shapes (Fig 1). Although the leaves displayed indefinite-and-varying shapes even within same varieties, these mature leaves could be classified into three basic types based on their modes of divarication (Fig 1). Wild-type leaves were not lobed (Fig 1A), but some varieties were bifurcated or trifurcated (or rather, had monopodial branching). For example, *M*. *pteropus* var. *windelov* (Fig 1B) and ‘*Gigantea*’ (Fig 1C) had bifurcated leaves, while leaves of ‘*Tropica*’ (Fig 1D), ‘*Thunder leaf*’ (Fig 1E), ‘*Fork leaf*’ (Fig 1F), and ‘*Trident*’ (Fig 1G) were monopodial. We investigated the genetic relationships among the varieties using a molecular phylogenetic analysis, based on a work of Kreier *et al*. (2008) [15]. Two species, *Platyceriu stemaria* and *Pyrrosia polydactyla*, were used as outgroups for constructing a phylogenetic tree, which included the *M*. *pteropus* wt, the six varieties, and other species of *Polypodiaceae* (Fig 2A and Table 2). The accession numbers of three genes (*rbcL*, *trnL−F*, and *rps4*) are shown in Table 2. Consequently, the wt and the six varieties were classified into one group. In this analysis, some relationships within or among each clade were unsupported; however, all the clades other than *Microsorium* included all species, as previously recognized. As strongly supported by Kreier *et al*. (2008) [15], the node including *Leptochilus* plus *M*. *pteropus* was demonstrated to be more distantly related to the nodes of *Microsorum* radical (asterisks in Fig 2A). Our analysis also revealed that the all seven varieties investigated, were included in the *M*. *pteropus* branch. We further examined the representative species marked with triangles in Fig 2A or Table 2, and fitted them into a phylogenetic tree (Fig 2B). When the wt and the six varieties were found to be more closely related to one another than the other species (Fig 2B). Therefore, we could assume that these plants were indeed closely related.

### Growths and divarications at each distal end of the leaves indicated in time-laps images

**Fig 3:**
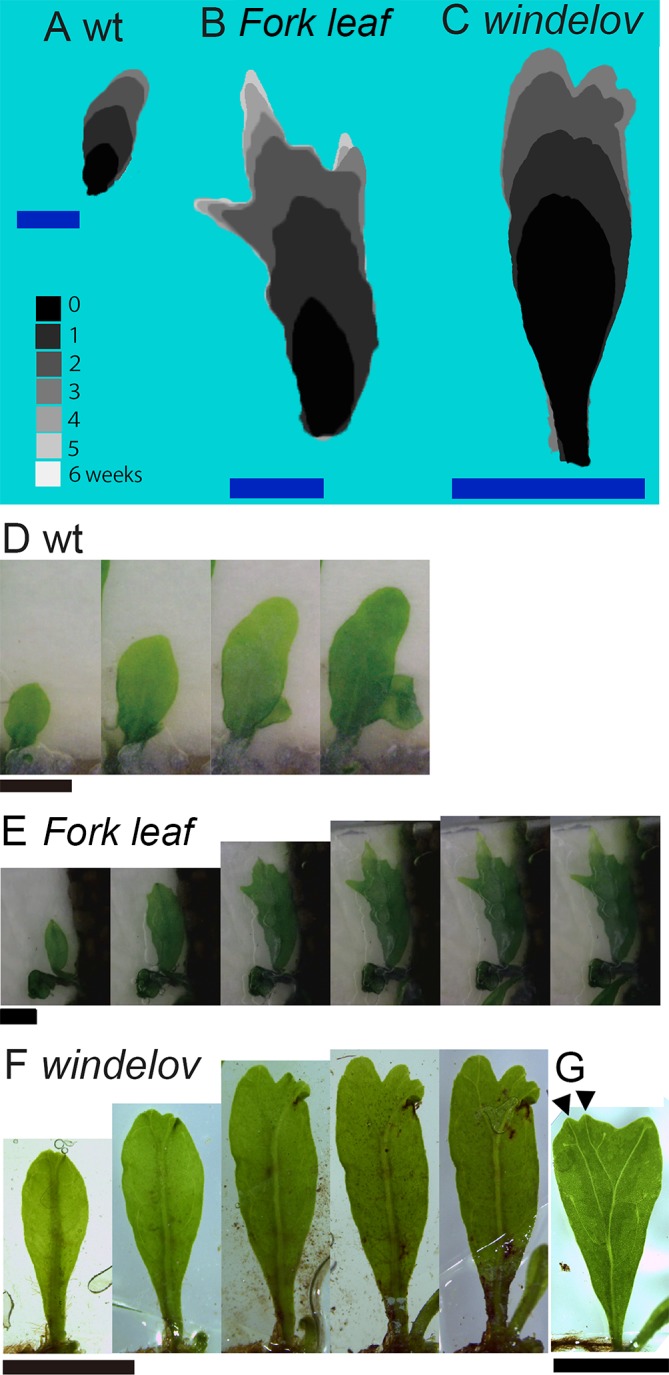
Time-lapse analyses of the different types of leaf divarication in *M*. *pteropus* and its varieties. Stacked silhouettes of representative types of growing leaves observed in *M*. *pteropus* cultivars (A–C). Obtained time-lapse images were stacked with silhouettes, with one-week-apart intervals (brightness of the gray scale images are assigned lighter hues over time). The color versions of each image are arranged from left to right in a time series (D–F): (A, D) *M*. *pteropus* wt, (B, E) *Microsorum sp. ‘Fork leaf’*, and (C, F) *M*. *pteropus* var. *windelov*. All the three types of leaves did not change in outline, positions of their trichomes, and leaf venation patterns, other than at each distal end. A blade bifurcation without a leaf vein bifurcation (G). Arrowheads indicate a recent blade bifurcation. All scale bars represent 5 mm.

Most leaves of ferns have coiled axes, or crosiers, during their early developments [12]. This attribute makes it difficult to observe the developments in the leaves before their expansions (i.e., later mature stages of the developmental sequence). The all leaves in the *M*. *pteropus* cultivars that we examined only had a small, coiled crosier (i.e., hook) for the short period before the expansion, and the leaves continued to develop and divaricate even after the expansions. Therefore, we could observe the leaf growths and the formations of characteristic leaf shape using a digital microscope (Fig 3). We acquired time-lapse images (200x magnification, four images per day for two months) from post-leaf expansions to cessations of the growths in the leaves of representative species with the three types of divarication. Weekly silhouettes of the images were stacked against each other using different shades of gray coloration (Fig 3A–3C). In the leaves of *M*. *pteropus* cultivars, growth terminations were irregular, and dissections of the blades occurred incidentally. The above attribute could be responsible for the indefinite shapes of leaf; however, each cultivar shared certain similarities. The leaf outlines did not change in the, time-laps images, except for the apices. When trichomes and leaf venations were overlapped at the base, they provided clear pictures of how the leaves grew from their apices. In both bifurcated and monopodial leaves, the blades diverged at each distal, growing part of the apices (Fig 3B, 3C 3E and 3F). A bifurcation of the leaf vein seemed to frequently precede a corresponding bifurcation of the blade. However, some bifurcations of the leaf vein did not accompany bifurcations of blade (Fig 3E). This phenomenon was often observed in *windelov* variants. Then a bifurcation of the blade without a bifurcation of the leaf vein could be observed (Fig 3G). In Fig 3G, a freshly bifurcated blades (arrowheads) had the leaf veins bifurcated at different times.

### Small epidermal cells and EdU labeled pairs of cells at each distal end of leaves

**Fig 4:**
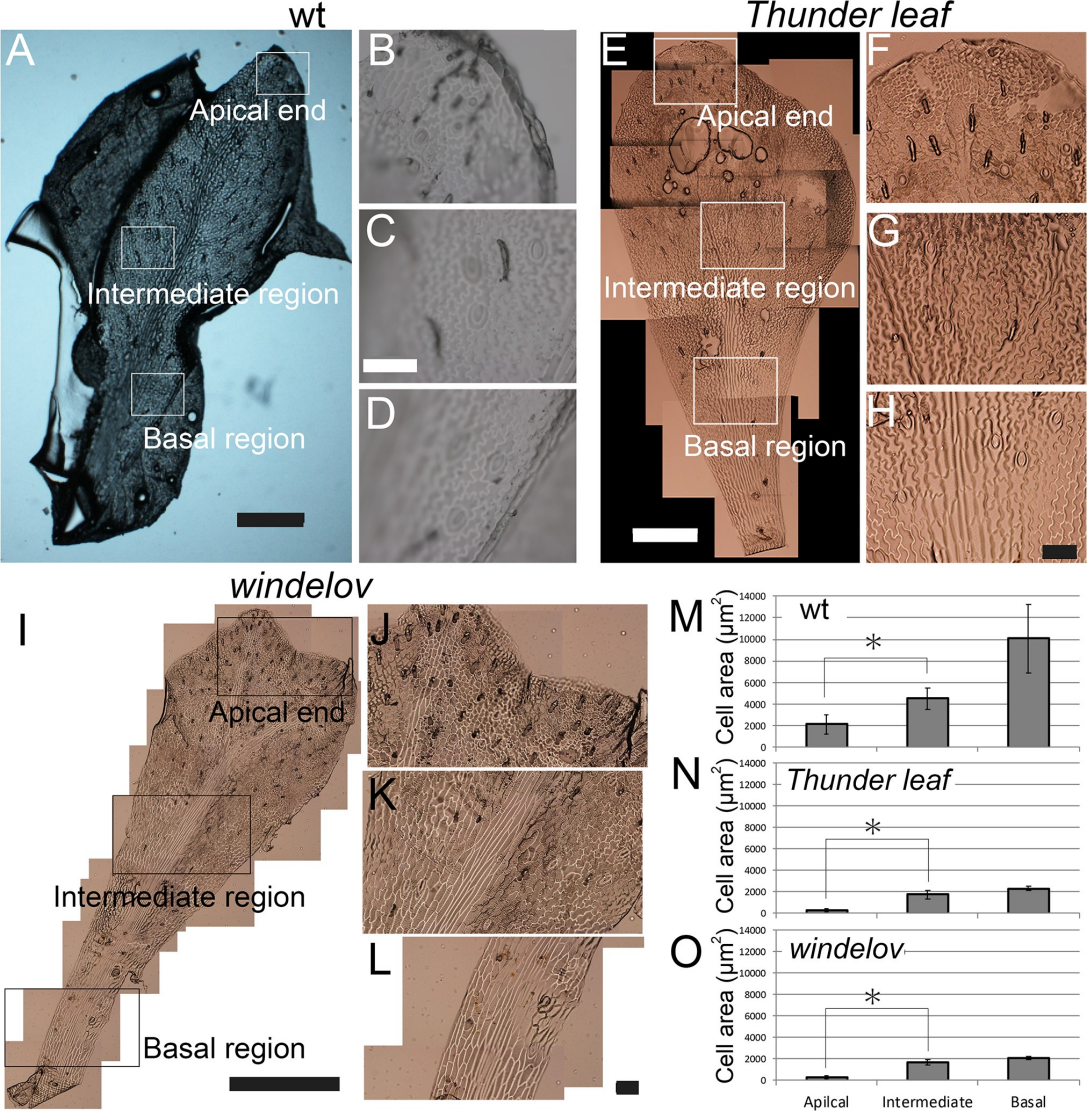
Sizes of epidermal cell on growing leaves in *Microsorum* varieties. (A–L) Microscopic images of epidermal cells on growing leaves of *M*. *pteropus* wt (A–D), *Microsorum* sp. ‘*Thunder leaf*’ (E–H), and *M*. *pteropus* var. *windelov* (I–L); (B–D, F-H, J–L) magnifications of each rectangular region in the left images. Images: (A, E, I) entire leaves, (B, F, J) apical ends, (C, G, K) intermediate regions, and (D, H, L) basal regions. (M–O) Cell sizes in the different regions of the leaves. (M) *M*. *pteropus* wt, (N) *Microsorum* sp. *‘Thunder leaf’*, and (O) *M*. *pteropus* var. *windelov*. *significant difference (p < 0.05) by Student’s t-tests. Scale bars represent 1 mm (A, E, I) and 100 μm (B–D, F-H, J–L).

In almost all leaves, cell expansion phases initiate after cell proliferation phases; then, differences in cell sizes and shapes can often be observed along the longitudinal axes [21], [22]. When we observed epidermal cells of developing leaves in *M*. *pteropus* and its varieties (using a replica method), we observed that simpler and smaller cells existed at each distal end. In contrast, larger pavement cells (having jigsaw-puzzle shapes) were located in more basal regions (Fig 4A–4L and S1 Fig). However, distances from the apices to regions of the smaller cells at the distal ends usually differed among the various types of *Microsorum* that we investigated. When we measured sizes of epidermal cell, the cells at distal end were always significantly smaller than the cells in more basal regions (Fig 4M–4O). From these results, we concluded that the all types of the investigated *Microsorum* leaves grew at each distal end, and cell enlargements follows via cell proliferations.

**Fig 5:**
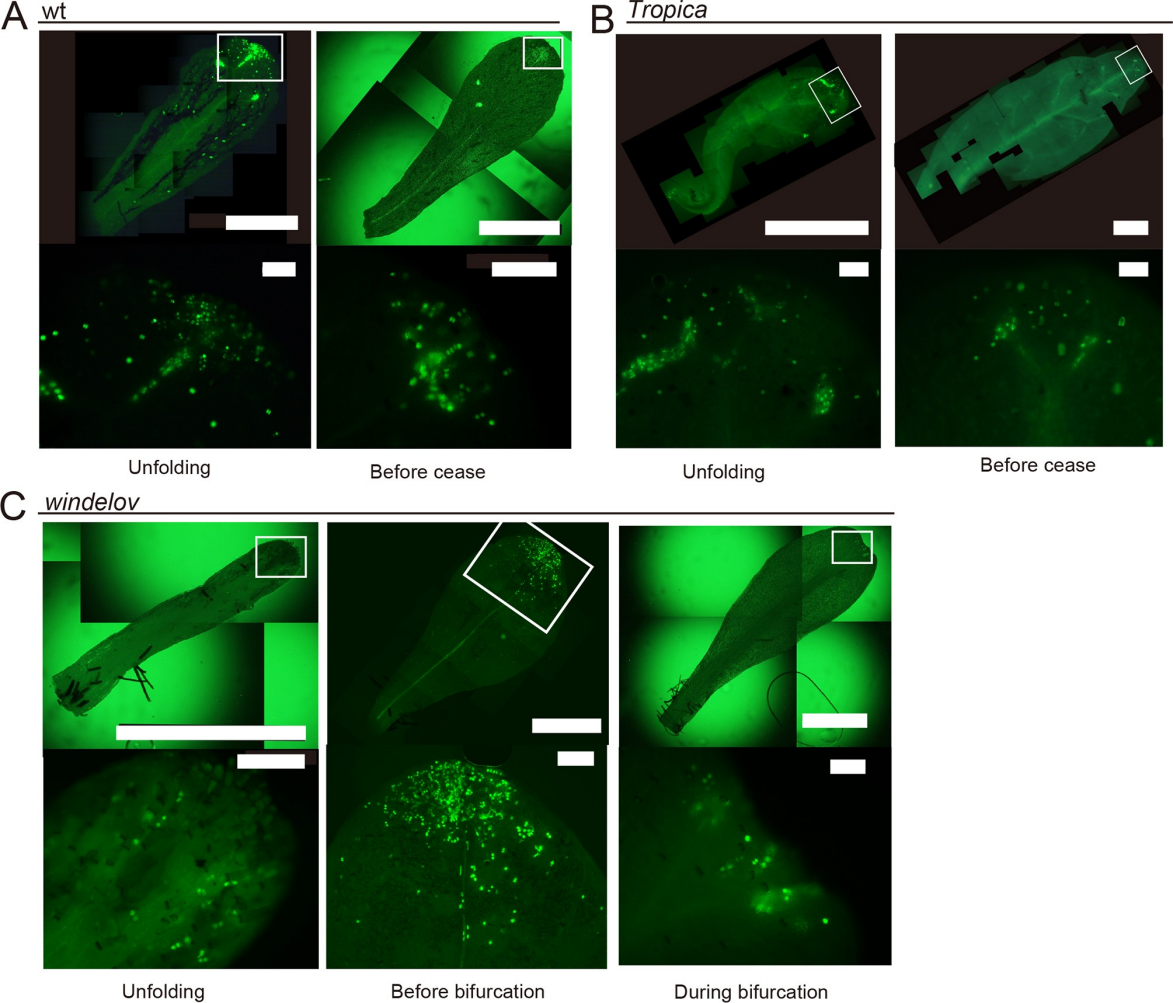
Pulse-chase analyses with EdU indicating the shifts of cell division sites according to leaf growth. Distributions of divided cells in leaves of (A) *M*. *pteropus* wt, (B) *M*. *pteropus ‘Tropica’*, and (C) *M*. *pteropus* var. *windelov* at two or three growth stages as visualized with EdU. The stages are indicated under the figures. In all sampled leaves, the signals were limited to each distal region (A–C). Lower panels indicate magnifications of each rectangular region in the upper images. The pair of green signals indicates divided daughter cells. Cell division sites were shifted to the distal end(s) based on growth-and-bifurcation patterns of the leaves. Scale bars represent 1 mm (upper panels) and 100 μm (lower panels).

Cell divisions in leaves of *M*. *pteropus* varieties were labeled using EdU, an analog of nucleoside. EdU labeling assays include signals obtained by endoreduplications, wherein cell cycles skip the mitotic phases [19]. The skipping seems to be typical for leaves in seed plants [23], [24]; however, there are few descriptions of endoreduplication in fern leaves [25]. To avoid signals obtained from endoreduplications, we used a pulse-chase strategy [9], [17], [18]. Consequently, almost all the labeled cells existed in each distal region of the leaf primordia in all investigated species, including all the three types and their branched versions (Fig 5 and S2 Fig). When the blades were branching, the labelled cells became included in each tip of the growing branches (Fig 5C and S2 Fig). We recognized that some of the labeled cells were divided, indicating presences of daughter-cell pair, at the tips of leaf blade and vein (Fig 5A–5C, lower panels). Apparently, the pattern of cell division shifted to more distal parts of the leaf based on the growth-and-divarication patterns of the leaves examined (Fig 5A–5C).

## Discussion

The investigated *M*. *pteropus* and its varieties were combined into one group based on a phylogenetic analysis (Fig 2). They exhibited a variety of leaf shapes, particularly in types of divarication, even among the closely related plants (Figs 1 and 2). In the time-lapse images that were taken, apical growths of the leaves were prominent (Fig 3). The smallest and simplest epidermal cells were observed in each distal part of the leaves (Fig 4 and S1 Fig). In the pulse-chase experiments (using EdU), fluorescently labeled pairs of daughter cells (i.e., indications of cell division) were detected only at the distal end(s) of the leaves (Fig 5 and S2 Fig). The cells were dividing in the limited regions, in where the apical growths leaf occurred. The data indicate that cells proliferate only at the distal part(s) of the fern leaves, which are updated continuously, then the cells expand on the site. Such apical growths would generate the bifurcated shapes characteristic in the fern species. Similar bifurcations in other plants could also be explained by such distal growths.

A bifurcation arises by an even splitting of a growth point at each distal end of a leaf, for example, during some long, continued apical-growth phases in ferns [12]. Conversely, two other types of divarication, which are common in plant species other than fern, would be also explained by other developmental patterns, such as basipetal, bidirectional, and diffuse growths. The monopodial branching occurs when new growth points are added to the lateral sides of a leaf.

A bifurcation of leaf veins seemed to frequently preceded a corresponding bifurcation of blade. However, our experiments revealed that some bifurcations of *Microsorum* leaf blade were not accompanied by the bifurcation of the leaf vein (Fig 3G). This phenomenon may further indicate that a splitting of marginal growth point precedes leaf vein bifurcation. The peripheral growth pattern in a leaf can be detected by accumulations of a phytohormone (i.e., auxin maxima) [1], [26], which induce leaf protrusions (e.g., lobes and serrations) and vein formations. From previous theoretical analyses, importance of such peripheral patterns in leaf morphogenesis has been proposed, however, major sites of cell divisions were in blades. Consequently, more detailed investigations are required to understand the interaction between the peripheral patterns and blade, and how variations in leaf shapes are produced. It has been demonstrated that directions of cell division plane are critical in shaping a leaf [21]. The relationship between the peripheral events of blade dissections and the cell division planes would also be interesting.

Branching patterns are not limited to plant leaves. Many other organisms display various branching patterns. For example, in three dimensional branches of mammalian lung morphogenesis, two primary forms of branching, a side branching and a tip bifurcation, were observed [27], [28]. Theoretical approaches for explanations of the difference between the branch patterns have been proposed [2], [29], [30]. The peripheral architectures of leaf have also been explored using mathematical models, with deformations of leaf margin based on a peripheral iterative pattern [1], [2]. All the various types of leaf divarication described in the present study can be obtained (Nakamasu unpublished 2019) using a same framework of the previously reported models [1], [2].

## Supporting information

S1 Fig Sizes of epidermal cell on a growing leaf in *M*. *pteropus “Tropica”*. (A–D) Microscopic images of epidermal cells on a growing leaf in *M*. *pteropus ‘Tropica’*. (B) Apical end, (C) intermediate region, (D) basal region. Scale bars represent 500 μm (A) and 100 μm (B-D).
(TIF)

S2 Fig Pulse-chase analysis with EdU indicating cell division sites in a branch of *Microsorum sp*. *“Thunder leaf”* leaf. (A) Distributions of divided cells in a *Microsorum sp*. *“Thunder leaf”* leaf with a branch at the tip. (B) The magnification of the rectangular region in the left image. Scale bars represent 500 μm (A) and 100 μm (B).
(TIF)
