# Supplementary figures and images for: Developmental analyses of divarications in leaves of an aquatic fern Microsorum pteropus and its varieties
Source: PLoS One. 2019 Jan 25;14(1):e0210141. doi: 10.1371/journal.pone.0210141 (PMC6347172; doi:10.1371/journal.pone.0210141)

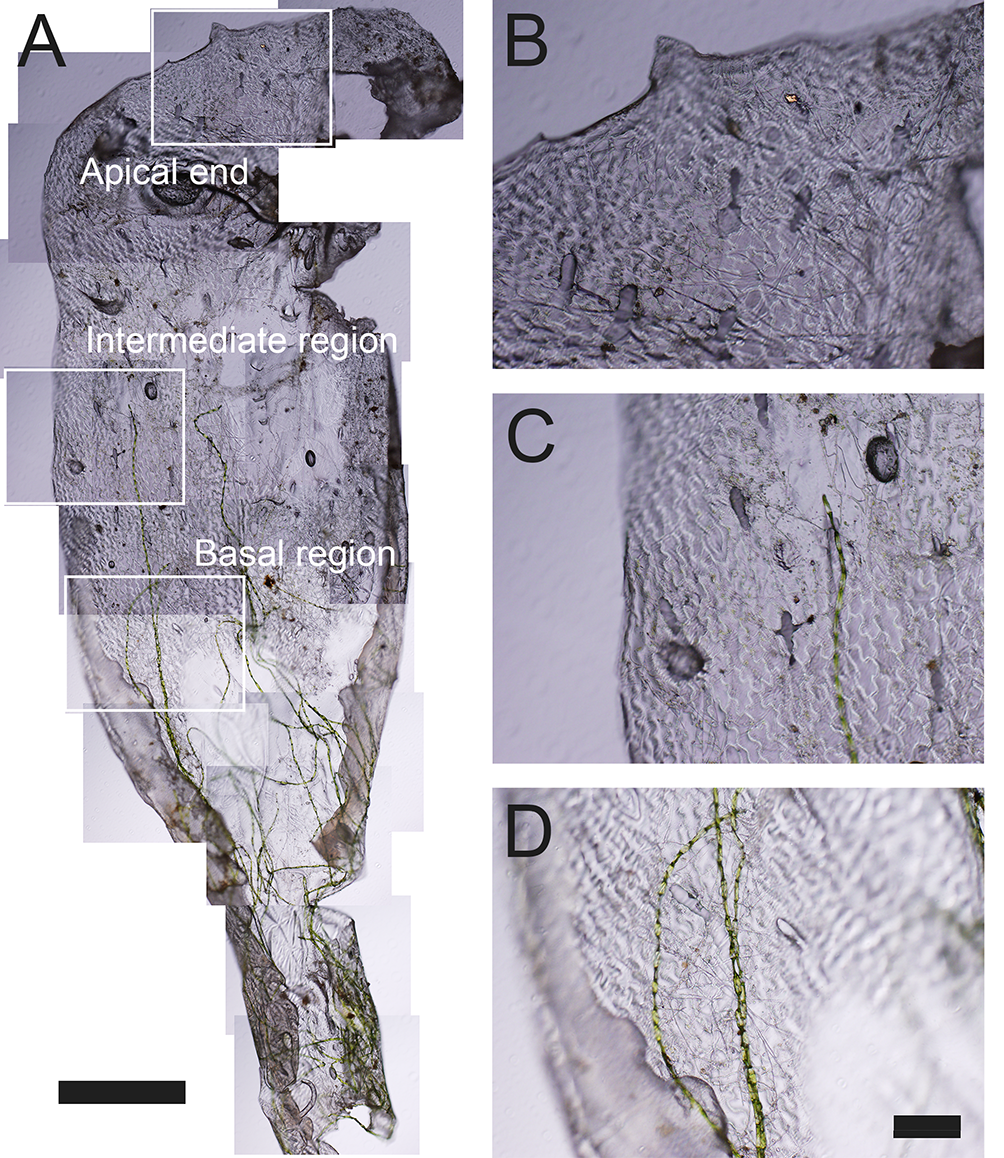

Supplement: S1 Fig — (A–D) Microscopic images of epidermal cells on a growing leaf in M. pteropus ‘Tropica’. (B) Apical end, (C) intermediate region, (D) basal region. Scale bars represent 500 μm (A) and 100 μm (B-D). (TIF) [file pone.0210141.s001.tif]

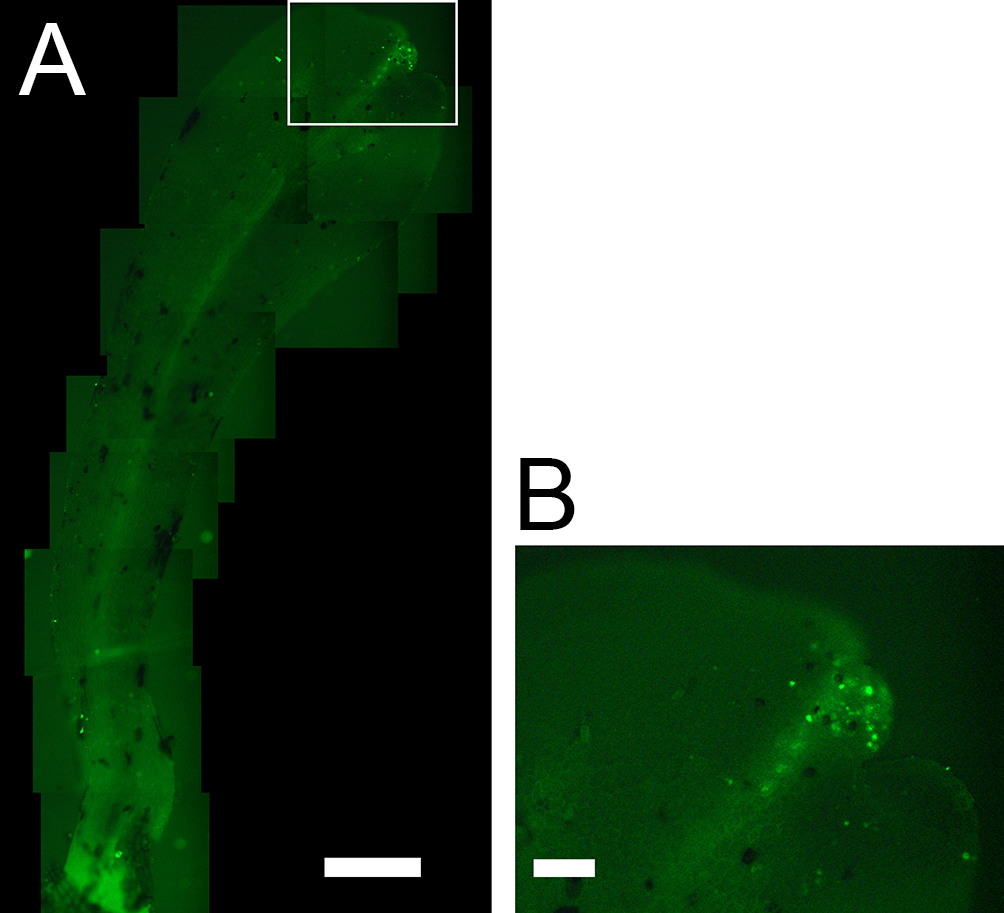

Supplement: S2 Fig — (A) Distributions of divided cells in a Microsorum sp. “Thunder leaf” leaf with a branch at the tip. (B) The magnification of the rectangular region in the left image. Scale bars represent 500 μm (A) and 100 μm (B). (TIF) [file pone.0210141.s002.tif]
